# Supplementary material for: Modeling glioblastoma heterogeneity as a dynamic network of cell states
Source: Mol Syst Biol. 2021 Sep 16;17(9):e10105. doi: 10.15252/msb.202010105 (PMC8444284; doi:10.15252/msb.202010105)
Supplement: Supplementary file 6 — Source Data for Figure 5 [file MSB-17-e10105-s004.zip › Figure5A_sourcedata/GSEA_3071/nosigdb_stateII.GseaPreranked.1622795151171/RICHARDS_NOWAKOWSKI_SCIENCE_2017_EN_PFC3_UPREG.html]

Details for gene set RICHARDS\_NOWAKOWSKI\_SCIENCE\_2017\_EN\_PFC3\_UPREG[GSEA]

|  || Dataset | state23071 |
| Phenotype | NoPhenotypeAvailable |
| Upregulated in class | na\_neg |
| GeneSet | RICHARDS\_NOWAKOWSKI\_SCIENCE\_2017\_EN\_PFC3\_UPREG |
| Enrichment Score (ES) | -0.5095048 |
| Normalized Enrichment Score (NES) | -2.9142916 |
| Nominal p-value | 0.0 |
| FDR q-value | 0.0 |
| FWER p-Value | 0.0 |
Table: GSEA Results Summary

  

Fig 1: Enrichment plot: RICHARDS\_NOWAKOWSKI\_SCIENCE\_2017\_EN\_PFC3\_UPREG      
 Profile of the Running ES Score & Positions of GeneSet Members on the Rank Ordered List

  

| PROBE | GENE SYMBOL | GENE\_TITLE | RANK IN GENE LIST | RANK METRIC SCORE | RUNNING ES | CORE ENRICHMENT || 1 | TLE3 |  |  | 215 | 0.861 | -0.0434 | No |
| 2 | TUBA1C |  |  | 277 | 0.779 | -0.0491 | No |
| 3 | ACAT2 |  |  | 446 | 0.653 | -0.0833 | No |
| 4 | SRM |  |  | 535 | 0.610 | -0.0979 | No |
| 5 | LIMCH1 |  |  | 552 | 0.602 | -0.0944 | No |
| 6 | NTM |  |  | 592 | 0.582 | -0.0970 | No |
| 7 | SMS |  |  | 608 | 0.575 | -0.0937 | No |
| 8 | TUBB |  |  | 678 | 0.538 | -0.1043 | No |
| 9 | NETO2 |  |  | 740 | 0.519 | -0.1133 | No |
| 10 | OCLN |  |  | 778 | 0.505 | -0.1163 | No |
| 11 | BASP1 |  |  | 802 | 0.497 | -0.1159 | No |
| 12 | CLMP |  |  | 822 | 0.490 | -0.1146 | No |
| 13 | RRP15 |  |  | 832 | 0.489 | -0.1108 | No |
| 14 | PRDM8 |  |  | 846 | 0.486 | -0.1081 | No |
| 15 | DERA |  |  | 886 | 0.474 | -0.1120 | No |
| 16 | ACTR3 |  |  | 909 | 0.469 | -0.1117 | No |
| 17 | NUAK1 |  |  | 1026 | 0.436 | -0.1355 | No |
| 18 | STX1A |  |  | 1060 | 0.426 | -0.1385 | No |
| 19 | RRAS2 |  |  | 1072 | 0.424 | -0.1360 | No |
| 20 | NT5DC3 |  |  | 1214 | 0.394 | -0.1666 | No |
| 21 | L1CAM |  |  | 1402 | 0.357 | -0.2093 | No |
| 22 | ZNHIT6 |  |  | 1524 | 0.336 | -0.2356 | No |
| 23 | SLC12A2 |  |  | 1712 | 0.309 | -0.2788 | No |
| 24 | VCAN |  |  | 1793 | 0.299 | -0.2952 | No |
| 25 | CACNA2D1 |  |  | 1878 | 0.289 | -0.3128 | No |
| 26 | FLRT2 |  |  | 2104 | 0.262 | -0.3662 | No |
| 27 | CAMK2N1 |  |  | 2111 | 0.262 | -0.3644 | No |
| 28 | PSMD11 |  |  | 2129 | 0.259 | -0.3655 | No |
| 29 | GNAI1 |  |  | 2254 | -0.254 | -0.3935 | No |
| 30 | DDX5 |  |  | 2257 | -0.255 | -0.3909 | No |
| 31 | ATAT1 |  |  | 2258 | -0.255 | -0.3877 | No |
| 32 | ZC2HC1A |  |  | 2277 | -0.257 | -0.3891 | No |
| 33 | SYT14 |  |  | 2296 | -0.258 | -0.3904 | No |
| 34 | SLC35E2B |  |  | 2297 | -0.258 | -0.3872 | No |
| 35 | NEUROD1 |  |  | 2312 | -0.261 | -0.3875 | No |
| 36 | KIF3A |  |  | 2315 | -0.261 | -0.3847 | No |
| 37 | PPFIA2 |  |  | 2364 | -0.266 | -0.3935 | No |
| 38 | SEMA6D |  |  | 2407 | -0.272 | -0.4007 | No |
| 39 | DTNA |  |  | 2414 | -0.272 | -0.3988 | No |
| 40 | EXOC4 |  |  | 2431 | -0.275 | -0.3994 | No |
| 41 | LMO3 |  |  | 2473 | -0.280 | -0.4063 | No |
| 42 | CLASP2 |  |  | 2514 | -0.287 | -0.4128 | No |
| 43 | FAT3 |  |  | 2526 | -0.290 | -0.4120 | No |
| 44 | TMEM108 |  |  | 2528 | -0.290 | -0.4086 | No |
| 45 | ZNF253 |  |  | 2552 | -0.294 | -0.4108 | No |
| 46 | SLC44A5 |  |  | 2557 | -0.294 | -0.4081 | No |
| 47 | SCN3B |  |  | 2595 | -0.300 | -0.4137 | No |
| 48 | MARCH6 |  |  | 2612 | -0.303 | -0.4140 | No |
| 49 | SORBS1 |  |  | 2639 | -0.307 | -0.4167 | No |
| 50 | TSPAN13 |  |  | 2706 | -0.318 | -0.4294 | No |
| 51 | CCDC28B |  |  | 2714 | -0.319 | -0.4272 | No |
| 52 | NHSL1 |  |  | 2735 | -0.324 | -0.4282 | No |
| 53 | CEP170 |  |  | 2737 | -0.324 | -0.4244 | No |
| 54 | SLC29A4 |  |  | 2741 | -0.325 | -0.4211 | No |
| 55 | MLLT11 |  |  | 2760 | -0.328 | -0.4216 | No |
| 56 | ATCAY |  |  | 2865 | -0.346 | -0.4435 | No |
| 57 | ELOVL6 |  |  | 2958 | -0.366 | -0.4621 | No |
| 58 | TMOD2 |  |  | 2985 | -0.370 | -0.4641 | No |
| 59 | ZNF286A |  |  | 2986 | -0.370 | -0.4595 | No |
| 60 | CALM1 |  |  | 2991 | -0.370 | -0.4559 | No |
| 61 | MIAT |  |  | 2996 | -0.371 | -0.4523 | No |
| 62 | TBL1XR1 |  |  | 3032 | -0.380 | -0.4564 | No |
| 63 | NDUFS5 |  |  | 3038 | -0.381 | -0.4529 | No |
| 64 | AKAP9 |  |  | 3066 | -0.389 | -0.4549 | No |
| 65 | CDH4 |  |  | 3164 | -0.418 | -0.4741 | No |
| 66 | FAM110B |  |  | 3191 | -0.426 | -0.4753 | No |
| 67 | RIMKLB |  |  | 3213 | -0.431 | -0.4753 | No |
| 68 | CHRDL1 |  |  | 3219 | -0.432 | -0.4712 | No |
| 69 | RTN1 |  |  | 3232 | -0.436 | -0.4688 | No |
| 70 | SOX5 |  |  | 3237 | -0.437 | -0.4643 | No |
| 71 | ZBTB18 |  |  | 3239 | -0.438 | -0.4592 | No |
| 72 | SSBP2 |  |  | 3251 | -0.442 | -0.4565 | No |
| 73 | HSBP1 |  |  | 3298 | -0.457 | -0.4624 | No |
| 74 | TSPYL1 |  |  | 3353 | -0.479 | -0.4700 | No |
| 75 | EIF1B |  |  | 3420 | -0.509 | -0.4803 | No |
| 76 | PPP1R14C |  |  | 3537 | -0.564 | -0.5025 | Yes |
| 77 | CTTNBP2 |  |  | 3541 | -0.565 | -0.4962 | Yes |
| 78 | GNG2 |  |  | 3543 | -0.567 | -0.4895 | Yes |
| 79 | TCEAL4 |  |  | 3552 | -0.572 | -0.4844 | Yes |
| 80 | TUBB2A |  |  | 3554 | -0.572 | -0.4775 | Yes |
| 81 | MAP4 |  |  | 3577 | -0.589 | -0.4758 | Yes |
| 82 | CRMP1 |  |  | 3583 | -0.594 | -0.4697 | Yes |
| 83 | NCOA1 |  |  | 3591 | -0.600 | -0.4640 | Yes |
| 84 | TRIM36 |  |  | 3602 | -0.605 | -0.4590 | Yes |
| 85 | FOXG1 |  |  | 3604 | -0.607 | -0.4517 | Yes |
| 86 | EPHA3 |  |  | 3616 | -0.614 | -0.4468 | Yes |
| 87 | CD24 |  |  | 3624 | -0.620 | -0.4409 | Yes |
| 88 | ITPR1 |  |  | 3627 | -0.624 | -0.4337 | Yes |
| 89 | KIF5A |  |  | 3631 | -0.627 | -0.4266 | Yes |
| 90 | DPYSL2 |  |  | 3645 | -0.642 | -0.4219 | Yes |
| 91 | SHC3 |  |  | 3648 | -0.645 | -0.4144 | Yes |
| 92 | BCL11B |  |  | 3656 | -0.651 | -0.4081 | Yes |
| 93 | ANK2 |  |  | 3657 | -0.651 | -0.4000 | Yes |
| 94 | DPYSL5 |  |  | 3668 | -0.659 | -0.3944 | Yes |
| 95 | TRIM2 |  |  | 3688 | -0.672 | -0.3908 | Yes |
| 96 | DUSP4 |  |  | 3745 | -0.724 | -0.3959 | Yes |
| 97 | BCL11A |  |  | 3756 | -0.735 | -0.3893 | Yes |
| 98 | GABARAPL2 |  |  | 3768 | -0.759 | -0.3827 | Yes |
| 99 | ENC1 |  |  | 3787 | -0.774 | -0.3776 | Yes |
| 100 | DAAM1 |  |  | 3856 | -0.849 | -0.3842 | Yes |
| 101 | GRIA3 |  |  | 3870 | -0.879 | -0.3765 | Yes |
| 102 | ZEB2 |  |  | 3876 | -0.894 | -0.3667 | Yes |
| 103 | IGSF3 |  |  | 3886 | -0.912 | -0.3576 | Yes |
| 104 | ROBO1 |  |  | 3920 | -1.005 | -0.3535 | Yes |
| 105 | PKIA |  |  | 3950 | -1.056 | -0.3477 | Yes |
| 106 | AFF3 |  |  | 3964 | -1.099 | -0.3373 | Yes |
| 107 | FRMD4B |  |  | 3972 | -1.116 | -0.3252 | Yes |
| 108 | SCG2 |  |  | 3975 | -1.137 | -0.3116 | Yes |
| 109 | TNIK |  |  | 3979 | -1.157 | -0.2980 | Yes |
| 110 | PGAP1 |  |  | 3981 | -1.164 | -0.2838 | Yes |
| 111 | CXADR |  |  | 3991 | -1.197 | -0.2712 | Yes |
| 112 | TTC28 |  |  | 3994 | -1.224 | -0.2565 | Yes |
| 113 | CSRP2 |  |  | 4000 | -1.238 | -0.2424 | Yes |
| 114 | GPM6A |  |  | 4002 | -1.248 | -0.2272 | Yes |
| 115 | TUBA1A |  |  | 4006 | -1.302 | -0.2118 | Yes |
| 116 | TUBB3 |  |  | 4009 | -1.334 | -0.1957 | Yes |
| 117 | PTPRO |  |  | 4011 | -1.344 | -0.1793 | Yes |
| 118 | DRAXIN |  |  | 4018 | -1.374 | -0.1637 | Yes |
| 119 | CHL1 |  |  | 4019 | -1.379 | -0.1466 | Yes |
| 120 | GAP43 |  |  | 4038 | -1.547 | -0.1320 | Yes |
| 121 | POU3F3 |  |  | 4049 | -1.649 | -0.1140 | Yes |
| 122 | PLXNA4 |  |  | 4060 | -1.752 | -0.0948 | Yes |
| 123 | POU3F2 |  |  | 4071 | -2.024 | -0.0722 | Yes |
| 124 | MEIS2 |  |  | 4073 | -2.041 | -0.0471 | Yes |
| 125 | LMO4 |  |  | 4074 | -2.051 | -0.0216 | Yes |
| 126 | NFIA |  |  | 4078 | -2.210 | 0.0050 | Yes |
Table: GSEA details [plain text format]

  

Fig 2: RICHARDS\_NOWAKOWSKI\_SCIENCE\_2017\_EN\_PFC3\_UPREG: Random ES distribution      
 Gene set null distribution of ES for **RICHARDS\_NOWAKOWSKI\_SCIENCE\_2017\_EN\_PFC3\_UPREG**
